# Cervical open-door laminoplasty technique with simple sutures and bone grafts: a single institutional study with 30 consecutive cases

**DOI:** 10.1186/s13018-015-0151-6

**Published:** 2015-01-28

**Authors:** Xin-Kui Li, Xu Liu, Lu Che, Chi-Jiao Ma, Dino Samartzis, Hai-Qiang Wang

**Affiliations:** Department of Orthopaedics, Xijing Hospital, Fourth Military Medical University, Xi’an, 710032 China; Aero Space Medical School, Fourth Military Medical University, Xi’an, China; Department of Orthopaedics and Traumatology, University of Hong Kong, Pokfulam, Hong Kong, SAR China

**Keywords:** Cervical spine, Laminoplasty, Surgery, Radiograph, Bone graft, Follow-up

## Abstract

**Background:**

Expansive open-door laminoplasty is widely accepted as a reliable procedure for cervical myelopathy. However, one acknowledged complication is spring-back complication or closure of the door which may result in restenosis of cervical canal and neurologic deterioration. The study aimed for addressing our cervical open-door laminoplasty technique with sutures and bone grafts and subsequently the follow-up outcomes.

**Methods:**

Thirty consecutive patients who underwent open-door laminoplasty with the novel technique were included and followed for minimum 5 years from Jan 2006 to Dec 2007. Anteroposterior diameter (APD) of the vertebral canal of C4 was measured in lateral cervical radiographs. Neurologic scenarios were noted using the Japanese Orthopaedic Association (JOA) scores.

**Results:**

Twenty-five males (83.3%) and five (16.7%) females with an average follow-up of 68 months were enrolled. The preoperative APD was 13.22 mm (±1.15), whereas the postoperative APD increased to 31.23 mm (±2.43) with an expansion ratio of 136.23% (*P* < 0.05). The JOA score increased from 8.5 preoperatively to 13.45 postoperatively with a recovery rate of 58.2% (*P* < 0.05). The elevated laminas were maintained open during the follow-up period.

**Conclusions:**

Our technique with sutures and bone graft for laminoplasty is a simple and efficient method for maintaining the decompression of cervical canal and neurologic improvement.

## Background

During the past decades, expansive open-door laminoplasty (EL) has been widely accepted and utilized as a safe and reliable treatment modality for multilevel cervical myelopathy. It has a number of advantages in long-term issues over laminectomy [1]. One expected advantage of EL over laminectomy is that it attains decompression and widening of the canal without removing posterior elements permanently, which results in expected better outcomes in the long-term run. Indeed, long-term follow-up lines of evidence shed light on the reliability of EL in terms of clinical and radiographic outcomes [2-6]. One the other hand, a multitude of studies addressed methodological modifications of the original EL reported by Hirabayashi [7], aiming for avoiding spring-back closure or collapse of the open door [8-19]. In our previous study, we noted that spring-back closure occurred as high as 10% by case and 6% by level [20].

These new treatment modalities aim for more valid fixation of the open door by introducing mini-plates, screws, anchors or clips. However, the implantation of the aforementioned hardware might render the procedures complicated and risky to certain extent. In an effort to find a simple and effective modality to maintain the canal expansion, we devised a modification of EL with simple sutures and bone grafts derived from removed spinous processes on the hinged side on the basis of computer-aided method as we previously reported [21]. This method allows for valid fixation of the elevated laminas without need of any additional instrumentation.

## Methods

The study was approved by our institutional ethics review board of Xijing Hospital, Fourth Military Medical Universtiy (No. 20050917_03), with written informed consent obtained from each patient. The study group consisted of 30 patients with cervical myelopathy who underwent cervical laminoplasty with sutures and bone grafts and were followed for minimum 5 years in a single institution from Jan 2006 to Dec 2007. All patients underwent cervical laminoplasty by a fixed and experienced spinal team (Prof. XK Li with 30 years of spinal surgical experience and Prof. HQ Wang with 10 years of spinal surgical experience).

### Operative technique

**Figure 1:**
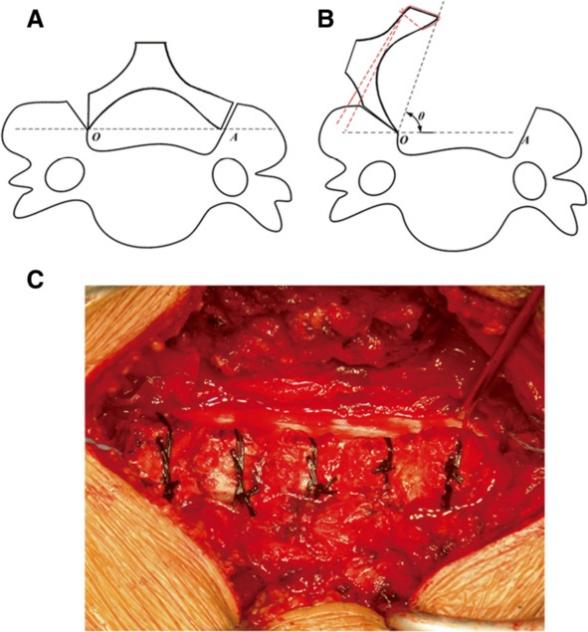
**Demonstration of the surgical technique. A**,**B** Schematic diagram showing the technique. The bone gutter is made and a hole is drilled on the lamina. The lamina is elevated with suture. In the panels, A represents decompression site; whereas O represents hinged site. θ represents the angle the lamina is evaluated. **C** Intra-operative picture delineates the surgical technique. C3-7 is elevated and fixed with suture.

The elevated laminas were determined according to the neurologic and radiographic status of the patients. The spinous processes of targeted laminas are cut short with an attempt to preserve interspinous ligaments and the facet capsules. The removed spinous processes are pruned into strips for bone grafts. Using an ultra-thin laminectomy rongeur, a gutter is created on the “open” side at the junction between the laminae and the facets. A thin-bladed Kerrison rongeur is used to remove ligamentum flava at the cranial and caudal ends of the intended laminar expansion, usually at the C2/3 and C7/T1 interspaces. Another gutter in the opposite side is made using a high-speed burr, whilst preserving the ventral cortex, which acts as a hinge. The stability of the hinge is checked frequently by applying a gentle bending force to the spinous processes to prevent hinge breakage. After the completion of the hinged gutter, laminas are elevated using a four-jaw clamp with a large Kerrison rongeur placed under the open edge to prevent the block of laminae to slip and rapidly snap back to original position, which may result in a spinal cord injury. A small hole is drilled using a high-speed burr in each open-sided lamina (Figure 1A). Sutures are placed through the facet joint capsules and surrounding soft tissues in the hinge side at each level and are subsequently passed through the hole of the corresponding elevated lamina. Strip bone grafts are placed under the sutures around the bone gutters of the hinged side. To maintain the expanded position and prevent the closure of the laminar door, sutures previously placed at the base of the spinous processes are securely tied (Figure 1B, C). Massive epidural bleeding might be encountered upon release of the particularly stenotic canal, which might be controllable with hemostatic material and gentle compression. The recovered pulsatile dura verifies satisfactory decompression.

Continuous drainage tube with negative pressure was used and the amount of drainage was monitored every 24 h following surgery. The drainage was removed once the amount of drainage was less than 50 mL within 24 h. Patients were immobilized with cervical collar for 4–6 weeks.

### Radiographic evaluation

**Figure 2:**
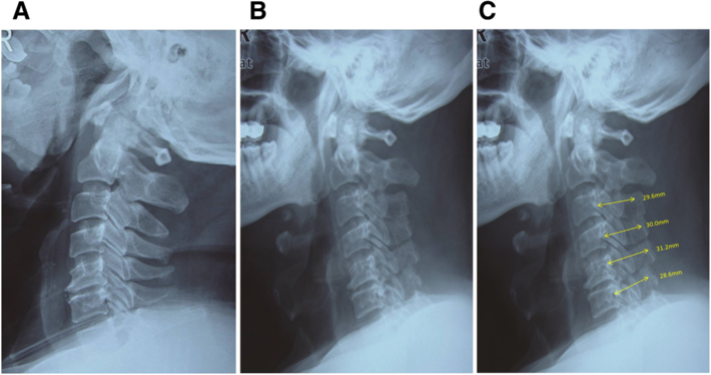
**Lateral radiographs showing expanded canal. A** Preoperative radiograph. **B** Immediate postoperative radiograph demonstrating elevated laminas. **C** Postoperative radiograph demonstrating the measurements of anteroposterior diameters of elevated laminas.

Series anteroposterior and lateral cervical spine radiographs were digitized and entered into a DICOM picture archiving and communication system (PACs). Visualization and measurements were performed with a Radworks 5.1 (Applicare Medical Imaging BV, Zeist, The Netherlands) system. The anteroposterior diameter (APD) of the cervical canal for C4 was measured in lateral cervical radiographs in PACs by two independent spine surgeons. Pre-operative APD was measured using Wolf’s method [22]. Post-operative APD was evaluated from the middle of the posterior border of the vertebral body to the anterior cortex of the elevated lamina as previously described [20] (Figure 2C).

### Neurologic outcome assessment

Neurologic status was evaluated using the Japanese Orthopaedic Association (JOA) scores for cervical myelopathy [23]. The JOA recovery rate was utilized to reflect the degree of postoperative recovery of normal function [1,6,7,24,25].

### Statistical analysis

All data were collected and noted on a spreadsheet and presented as mean ± SD. SPSS 17 (Chicago, IL, USA) package was used to perform the statistical analyses. Inter-class coefficient (ICC) was used to evaluate inter-rater reliability. Accordingly, reliability scores of less than 0.79, 0.80 to 0.89, and greater than 0.90 were considered as poor, good and excellent, respectively [26]. ANOVA with repeated measures before and after surgery was compared. The threshold of significance was set as *P* value <0.05.

## Results

There were 25 males and 5 females with an average age of 58.7 years (range 30–73 years). The mean postoperative follow-up was 68 months. The causes of the myelopathy were the ossification of the posterior longitudinal ligament in four patients and multilevel spondylosis in 26 patients. Eleven patients had symptoms of radiculopathy. Six patients presented due to injuries which aggravated symptoms of cervical stenotic myelopathy. The average duration of cervical myelopathy prior to presentation was 26.9 months. In total, 124 laminas were elevated. The mean number of lamina elevated was 4.1 for each patient. Levels of laminoplasty were as follows: C3-6 in 26 patients and C3-7 in 4 patients.

### General outcome

In general, all patients experienced decreased numbness of limbs and increased muscular strength following surgery. There were no cases with postoperative hematoma. The mean operative time was 1.5 (±SD, 0.25) h. The mean blood loss was 315 (±SD, 21) mL intraoperatively, whereas the amount of drainage postoperatively was 195 (±SD, 11) mL.

### Radiographic outcome

**Figure 3:**
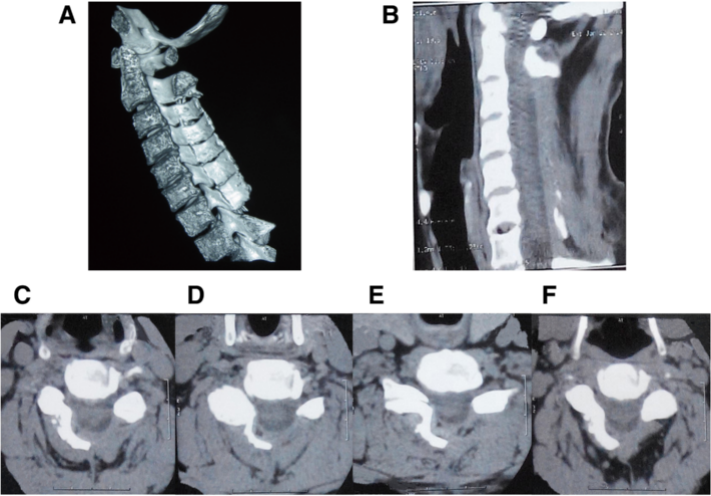
**Representative CT images. A** Three-D CT demonstrating expanded canal. Six-year postoperative 3-D CT with canal expansion maintained. **B** One-year postoperative sagittal CT image of a 62-year-old male. **C–F** Axial CT images of the case corresponding C3-4, C4-5, C5-6 and C6-7.

**Figure 4:**
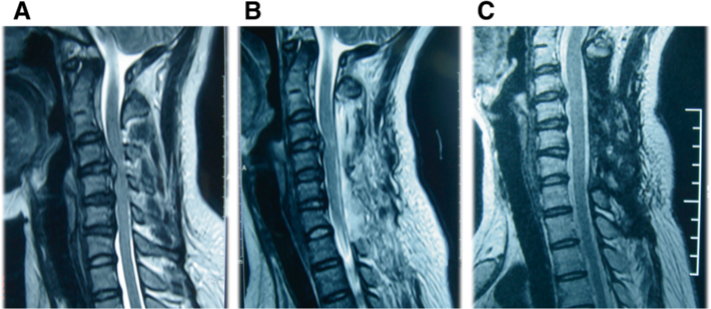
**Series MRI of a female patient undergoing laminoplasty with the novel technique. A** Preoperative MRI. **B** Immediate postoperative MRI demonstrating canal expansion with huge herniated C4/5 disc. Note the posterior shift of cervical spinal cord. **C** Five-years postoperative MRI demonstrating the vanished herniated C4/5 disc with improved neurologic manifestation.

The APD measurement was noted as high inter-rater reliability with ICC > 0.90. The preoperative APD was 13.22 mm (±SD, 1.15), whereas the postoperative APD increased to 31.23 mm (±SD, 2.43) with an average expansion ratio of 136.2% (*P* < 0.05, Figures 2, 3 and 4). No cases were noted with spring-back closure. The elevated laminas were maintained open during the follow-up period.

### Neurologic outcome

The mean preoperative JOA score was 8.5 (±SD, 2.9). At final follow-up, the postoperative JOA score was 13.45 (±SD,3.4) with a recovery rate of 58.2% using the method as previously proposed [7] (*P* < 0.05). One patient experience postoperative transient C5 motor paresis and eventually recovered within 6 months.

## Discussion

Cervical laminoplasty provides an alternative for multisegmental canal stenosis. It should be stressed that keeping the expansion obtained from laminoplasty is of crucial importance for the surgery. Postoperative deterioration due to spring-back closure of elevated laminas has been noted as a critical complication [1,3,20]. Indeed, Hirabayashi et al. noted re-closure of the door and utilized the supporting sutures to avoid it [1,7]. The concern of such complication has led numerous investigators to explore a variety of modified methods. Amongst the different modified methods, simplicity and efficiency are the ultimate pursue. However, the employment of plates, screws, anchors and clips might counteract the simplicity and increase the operation time, blood loss and morbidity. To address these issues, we devised the novel cervical laminoplasty with simple sutures and bone grafts to maintain the door open.

As a result, none of the patients in the study was noted with spring-back closure. The door kept open at the final follow-up period. Furthermore, we could attain satisfactory canal expansion up to 136.2%. Studies have shown that improvement directly correlates with the degree of canal expansion [1,27]. On the basis of the current available literature, we compared the expansion ratios in the literature (Table 1). From this point of view, our technique with simple sutures might achieve the maximum canal expansion ratio according to the comparison.

**Table 1:** **APD increments and expansion ratio after laminoplasty in the literature**

	**Technique**	**Radiography**	**Increments or expansion ratio at final follow-up**
Hirabayashi [1] 1983	EL	PLR	Mean increase 3–4 mm
Hirabayashi [23]1999	EL	PLR	4.4 mm
Chiba [6] 2006	EL	PLR	4.4–5.9 mm
Satomi [2]1994	EL	PLR	3.0 mm
Yang [16] 2007	Suture anchor fixation	CT	38.4–54.3%
Baba [18] 1997	En bloc	3D CT	Mean increase 42%
Baba [23] 1995	Tomita	CT	6.8 mm 40%
O’Brien [8] 1996	Titanium		
Miniplate	CT	8.4 mm 105%	
Wang [10] 1998	Anchor system	PLR	6.3 mm 64%
Itoh [19] 1985	Bone blocks with wire ligatures	PLR	4.1 mm
This study	Simple sutures	PLR	8.01 mm 136.23%

EL: Expansive open-door laminoplasty; PLR: Plain lateral radiograph; CT: Computed tomography.

In addition, the cervical canal increments obtained by the classic Hirabayashi EL range from 3 to 5.9 mm according to various studies [1,2,6,25]. A number of studies with modified techniques hardly break through the ranges [10,16,18,19,27]. Amongst the reported modified techniques, O’Brien and colleagues noted a novel laminoplasty using miniplate augmentation with an expansion ratio of 105% [8]. Their technique is also noteworthy, owing to the similar expansion ratio with ours. Consistent with our study, they also noted vigorous epidural bleeding upon decompression, which has been scarcely noted by other authors. This might be due to sufficient canal expansion and complete decompression upon the stenotic canal. However, the bleeding is totally controllable. On the other hand, the employment of plates and screws in O’Brien’s technique might weaken the advantages of the procedure. In particular, our simple sutures technique rather than plates augmentation is relatively simple.

Since the introduction of anterior cervical discectomy and fusion (ACDF) by Smith and Robinson in 1958, it has been widely performed to treat patients with degenerative cervical diseases. However, controversy still exists regarding the appropriate surgical treatment for multi-segmental degenerative cervical diseases involving three or more segments. For single or two-level cervical degenerative disease, we routinely choose ACDF with autogenous iliac bone graft and plate. For multi-segmental cervical degenerative disease over three segments or with cervical stenosis (as the case in Figure 4), we usually choose cervical expansive laminoplasty as we reported in the study on the basis of several factors. First, it will be very difficult, if not impossible to harvest over 3 suitable iliac bone stamps for reconstruction of dissected discs. Second, the technique we reported is economic in comparison with ACDF since no instrumentation is needed. No matter which approach the surgeon chooses, improved clinical outcome with ameliorated symptoms and limited costs.

Despite our study presented a novel open-door laminoplasty with relatively satisfactory outcomes, several limitations were present. The study comprised a relatively small number of patients and belonged to the type of retrospective study in essence. However, the technique and our experience in its attendant utilization can provide some beneficial information with regard to cervical laminoplasty surgical strategies.

## Conclusions

In summary, we present our modification of laminoplasty with simple sutures and bone grafts. On the basis of our clinical and radiographic results, we find that the method is simple and effective. Furthermore, the method might attain the maximum canal expansion ratio according to the literature.
